# A socio-ecological analysis of barriers to the adoption, sustainablity and consistent use of sanitation facilities in rural Ethiopia

**DOI:** 10.1186/s12889-017-4717-6

**Published:** 2017-09-13

**Authors:** Fikralem Alemu, Abera Kumie, Girmay Medhin, Teshome Gebre, Phoebe Godfrey

**Affiliations:** 1 Ethiopian Institute of Water Resources, Water and Public Health program, Addis Ababa University, Addis Ababa, Ethiopia; 2 School of Public Health, College of Health Sciences, Addis Ababa University, Addis Ababa, Ethiopia; 3 Aklilu Lemma Institute of Pathobiology, Addis Ababa University, Addis Ababa, Ethiopia; 4 International Trachoma Initiative, Addis Ababa, Ethiopia; 5 Department of Sociology, University of Connecticut, Storrs, USA

**Keywords:** Sanitation, Ethiopia, Rural, Barriers, Adoption of latrine, Use of latrine

## Abstract

**Background:**

Despite evidence showing that access to and use of improved sanitation is associated with healthier households and communities, barriers influencing the adoption and sustainablity of sanitation facilities remain unclear. We conducted a qualitative case study to explore barriers influencing the adoption, sustainablity and consistent use of sanitation facilities in rural Ethiopia.

**Methods:**

A qualitative study was conducted in the rural district of Becho, in central Ethiopia, from June to August 2016. A socio-ecological model and Integrated Behavioural Model (IBM) for a Water Hygiene and Sanitation (WASH) framework were employed to design the study and analyse data. A total of 10 in-depth interviews (IDI) were conducted with latrine adopters (*n* = 3), latrine non-adopters (n = 3), health extension workers (n = 3) and the district WASH coordinator (*n* = 1). Eight Focus Group Discussions (FGD) were undertaken with 75 participants, of which 31 were women. The FGDs and IDIs were tape-recorded, transcribed verbatim and translated into English. The analysis was supported using Nvivo version 10 software.

**Results:**

Barriers to sustained adoption and use of sanitation facilities were categorized into 1) individual level factors (e.g., past latrine experience, lack of demand and perceived high cost to improved latrines), 2) household level factors (e.g., unaffordability, lack of space and absence of a physically strong family member), 3) community level factors (e.g., lack of access to public latrines, lack of shared rules against open defecation, lack of financial access for the poor), and 4) societal level factors (e.g., lack of strong local leadership, flooding, soil conditions, lack of appropriate sanitation technology, lack of promotion and demand creation for improved latrines).

**Conclusion:**

The use of the socio-ecological model and IBM-WASH framework helped to achieve a better understanding of multi-level and multi-dimensional barriers to sustained latrine adoption. The results indicate that there is a need to consider interventions that address multi-level factors concurrently.

## Background

Access to safe and adequate sanitation is a basic human right, affects health and the environment and is also an economic concern [1–3]. The WHO/UNICEF Joint Monitoring Programme (JMP) categorizes the types of sanitation facilities used by household members and presents sanitation as a four-step ladder: open defecation (the bottom of the ladder) and unimproved, shared, and improved sanitation facilities (moving up the ladder and reflecting progress) [4].

Evidence shows that access to and use of improved sanitation is positively associated with healthier households and communities [5–7]. The use of improved sanitation reduced the incidence of diarrhoeal diseases by 15–36% [3, 5, 7]. However, in 2015, more than 2.4 billion people globally lacked access to improved sanitation facilities, nearly all of them in developing countries [8]. Approximately 1.7 billion cases of diarrhoea occur every year, claiming the lives of approximately 800,000 children below 5 years of age [7, 9].

Despite all efforts, sanitation strategies and programmes in developing countries have often been challenged by the emerging complex nature of individual, social and environmental barriers [10]. An in-depth exploration and analysis of the multi-level factors at play in the adoption and consistent use of sanitation facilities are crucial to fully understand enabling and limiting contextual and technology-related factors.

Although Ethiopia has made remarkable progress in decreasing open defecation from 82% in 2000 to 29% in 2015, an estimated 72% of the population still lack access to an improved latrine [8]. Infectious and communicable diseases account for approximately 60 to 80% of the health problems in the country and diarrhoea is the second leading cause of death among children under 5 years of age [11], with no progress over a decade [12–14]. Subsequent efforts have been made by government and international partners to improve sanitation in Ethiopia. The rural Health Extension Programme (HEP) that was launched a decade ago has mainly focused on hygiene and sanitation promotion at the household level [15]. Community-Led Total Sanitation (CLTS), which is a participatory behavioural-change approach to rural sanitation, was introduced in Ethiopia by NGOs in 2007; late in 2011, CLTS was adopted as the country’s national hygiene and sanitation promotion strategy, with an added hygiene promotion (H) component [16]. It has been scaled up in rural Ethiopia since 2013. The national sanitation marketing guideline was developed in 2013 [17] and a pilot sanitation marketing project was recently implemented and is currently in the initial stages of development [18].

Researchers have suggested that interventions targeting multiple levels bring more sustainable change than individual-based interventions from a single-level analysis [19]. The recently published literature review, entitled “Sustainable Sanitation for All,” argues that a wide range of factors influence sustainability, including enabling the environment, physical conditions, leadership, programme quality, and an individual’s financial capacity [20]. Prior studies in Ethiopia and in other African countries show that having a better education, a larger family, and being male were significantly associated with the increased adoption and use of latrines [21–27]. Qualitative studies on sanitation have reported that the unaffordability of building materials [28–30] and the lack of awareness of the health risks of open defecation were barriers to the adoption of sanitation facilities [25, 30]. These studies have increased our knowledge of and the relationship between the factors that influence the adoption and use of sanitation facilities [21–26]. However, most of the studies focused on a single-level analysis [21–26]. Many of the behavioural, contextual and technology-based factors at various levels that might vary according to time and place have not been well documented.

The socio-ecological model (SEM) is widely recognized as providing a comprehensive approach for analysing and addressing multiple levels of influences. The model explains that a specific behaviour is influenced by factors that exist at the intrapersonal, interpersonal, community, and societal/policy levels and that there can be interaction of factors across levels [31–33]. The integrated behavioural model (IBM), which was derived from the SEM, was developed to guide research and interventions in the field of water, sanitation and hygiene (WASH) [10]. According to the IBM-WASH model, psychosocial, contextual and technological dimensions operate and interact at the societal/structural, community, interpersonal/household, individual and habitual levels. The current study applied SEM and a modified IBM-WASH model (habit is regarded as an individual-level psychosocial factor) for the design and analysis of data to understand factors contributing to or hindering sustained adoption and consistent use of sanitation facilities. It is believed that the current study will contribute to informing sanitation strategies for increased latrine adoption, latrine use and movement upwards on the sanitation ladder.

## Methods

### Study setting

The Oromia Region, where the study was conducted, is one of Ethiopia’s nine federal regional states. Becho *woreda* (district) is located 80 km southwest of Ethiopia’s capital, Addis Ababa. Based on the 2007 national census, Becho had a projected total population of 88,550 people in 2016, 80.4% of them rural residents [34].The livelihood of Becho *woreda* residents is categorized as mixed farming, and the main economic activities are crop and livestock production. The major crops produced in rural Becho are teff, wheat and chickpeas. Becho has a black cotton soil with very low permeability [35]. The majority of people in the area are Orthodox Christian by religion and from the Oromo ethnic group. Administratively, the district is divided into *kebeles* (*n* = 21) and each kebele is divided into a *gott*, where gott is the lowest strata. A network of community members, called the “Health Development Army (HDA)”, organized by the government, aims to discuss and solve their own social, security and health problems, including sanitation. Each group comprises up to 30 households residing in the same neighbourhood, and each HDA group is further divided into smaller groups of six members, commonly referred to as one-to-five networks. Two health extension workers (HEWs) have been assigned per *kebele* (the smallest administrative unit in Ethiopia) since 2005 and have been providing health education on a door-to-door basis [15]. CLTSH has been implemented in the area since 2013 as part of the scale-up in rural Ethiopia. According to the *woreda* report, CLTSH-triggering was conducted in 36 villages, reaching 17% of the rural villages in the district. However, only three declared themselves ODF (Open-Defecation Free) and three of them slipped back to Open Defecation (OD). CLTSH-triggering was conducted in all the study *kebeles.*

### Study design and samples

We conducted a qualitative study employing focus group discussion (FGD) and in-depth interview data collection techniques (Table 1). Six out of nineteen rural *kebeles* were randomly selected using a lottery sampling technique. Initially, the *kebeles* were stratified into ‘high’ latrine coverage and ‘low’ latrine coverage based on their performance in 2015, and three *kebeles* from each stratum were randomly selected. A list of households of the selected *kebeles* were obtained from HEWs. To explore the individual and household-level factors, three latrine adopter household heads and three non-adopter household heads were randomly selected using a lottery method from the list of households of selected *kebeles*. To explore the individual and community-level factors, eight focus group discussions (FGDs) were undertaken with 75 participants who were randomly selected from the study *kebeles*. To maintain group homogeneity and to encourage open discussion, FGDs were held separately with adult men, adult women, boys and girls (ages 15 to 18). Participants of FGDs were also stratified into two groups based on latrine ownership. Two FGDs were held with latrine adopter household heads, and two FGDs were held with latrine non-adopter household heads. Participants for FGDs were recruited by the local village leaders and the HEW. To explore societal-level factors, key informant interviews were held with one district WASH coordinator and three HEWs that were selected purposively*.* Two HEWs were from lower latrine-coverage *kebeles*, and one HEW was from a higher latrine-coverage *kebele*.

**Table 1:** Socio-demographic characteristics of study participants

No	Data collection type	No. of FGD/IDI	No of participants		
			Male	Female	Total
1	IDI with adopters	3	2	1	3
2	IDI non-adopter	3	2	1	3
3	KII with HEW	3	0	3	3
4	KII with WASH coordinator at district	1	1	0	1
5	FGD with adult women	1	0	10	10
6	FGD with adult men	1	10	0	10
7	FGD with youth(female)	1	0	9	9
8	FGD with youth(male)	1	10	0	10
9	FGD with non-adopters	2	11	7	18
10	FGD with adopters	2	13	5	18
	Total	15	49	36	85

*IDI* In-depth interview; *KII* Key informant interview; *WASH* Water, Hygiene, and Sanitation; *FGD* Focus group discussion; *HEW* Health Extension Workers

### Data collection procedures

Open-ended topic guides for IDI and FGD were designed and guided by the SEM and the IBM-WASH framework, while some specific questions were adopted from previous studies [26, 36]. Topics covered included current latrine-use practice, past exposure and experience with latrines, awareness and child-faeces-disposal practice, beliefs and perceptions towards latrines; contextual and technology-related barriers for latrine adoption, sustainability, and consistent use. The topic guides were prepared in English and translated into the Oromiffa language. The translated versions were used for the IDIs and FGDs. All IDIs and FGDs were conducted in Oromiffa, the local language of the study area, and were tape-recorded. The first author (FA) conducted all the FGDs and IDIs, while two Oromiffa speakers, with an education-level diploma, co-facilitated the FGDs. On average, an IDI lasted 45 min, while an FGD took 60 min.

### Data management and analysis

All IDIs and FGDs were transcribed into Oromiffa while being recorded. The transcribed documents were translated into English in a meaning-based approach. The first author (FA) translated all transcripts into English, and the second authors (AK and TG) read and provided feedback on all translations. Content analysis and thematic coding were performed and supported by NVivo 10.0 software (QSR International Pty Ltd.). The first author (FA) and one of the study team members (BY) coded the initial four transcripts independently, and the other co-authors reviewed the coding. Any differences were discussed until consensus was reached, and adjustments were made to the coding. All subsequent transcripts were coded by the FA. When new themes were identified, they were brought to the research team, and consensus was reached after thorough discussion. Texts that represented the codes were then quoted to validate the findings. The thematic categories were further examined to determine whether they fit the concepts, propositions and theories under consideration (the SEM and IBM-WASH framework).

### Quality assurance

Several measures were taken to ensure data quality. This study was designed after reviewing the relevant literature. The principal investigator remained at the study site for over a month before designing the study. Moreover, there were continuous debriefing sessions among members of the research team during data collection and data analysis. Data were collected from various sources for triangulation of information and to ensure conformability. A description of the context in which the study took place was provided so that transferability could be based on an adequate understanding of the study settings. All the collected data, including tape recordings, transcripts, and field observation notes, were documented and stored in a secured, locked cabinet.

### Ethical considerations

Ethical approval was obtained from the Ethical Review Board of the Oromiya Regional Health Bureau. Before beginning the interviews, data collectors were provided with detailed information about the objective of the study, on how to manage the information sheet and how to obtain a consent form. Written informed consent was obtained from each respondent. The consent was presented orally whenever the participant was unable to read or was reluctant to do so. In a situation where consenting participants could not read or write, they made a thumbprint on the informed consent. Parental consent was obtained for participants from ages 15 to 18. The IDIs were held in a private place at participants’ homes, and FGDs were conducted in the village meeting places close to those homes. Confidentiality was ensured throughout the process.

## Results

A total of 85 individuals with a mean age of 33.7 years participated in the study, and 57.6% were males. In terms of age categories, 22.4% were below 20 years, 16.5% were between 20 and 29 years, 11.7% between 30 and 39 years, 24.7% between 40 and 49 years, and 5.9% were over 49 years of age. Among the study participants, 44.5% were latrine non-adopters, 70.5% were married, 9.0% were widowed/divorced, 20.0% had never married, and all participants were from the Christian religion and the Oromo ethnic group.

### Adoption and consistent use of latrines

Open-defecation practice has decreased remarkably over the years. However, partial use of latrines emerged as a major challenge arising from daily routines, which take individuals away from their homes. Adult men practise open defecation during the daytime while they work on farms away from home. Adult women and young boys and girls use latrines because they have access to them in their home or at school. When infants defecate in their clothes, their caregivers wash the clothes and throw the dirty water outside the compound. Children from one to six years old defecate on the ground in the household compound, and their caregivers pick up the faeces with an *akafa* (spade) and dispose of it in the latrine. Some participants reported throwing children’s stool outside the compound.

### Individual psychosocial factors

Positive beliefs about latrine use were expressed by latrine adopters as well as by non-adopters. Disease prevention and fewer expenditures on health care were frequently cited as benefits of latrine adoption. Participants explained how the faeces from open defecation contaminate their food through flies, which they learned mostly from CLTSH-triggering exercises. However, there was also a perception of minimal health threat from children’s faeces, and a belief that they cause only minor illnesses or affect only small children.

The use of a latrine was regarded as attractive in terms of privacy, as being seen while defecating is often regarded as a shameful and unsightly behaviour in the community. Most men stated that protection of their family’s safety and dignity was their motivation for deciding to build their own latrine. Concerns such as women being raped or attacked by an animal when they go into the bush during the night, or small children falling into the fire due to the absence of supervision when their mother goes out for open defecation, were motivations for building a latrine.

"*Protecting my family is the main reason for my decision to build a latrine. My wife and my daughter can be attacked by a hyena or other animals or might get raped whenever they go to the field for open defecation at night. My kids can be at risk of getting burned or other accidents whenever my wife leaves them to go to the open field for defecation."*(FGD 7, 42-year-old male adopter).

Cleanliness was also one of the most valued benefits of latrine use, mostly mentioned as beautifying their surroundings. Some FGD participants noted that latrine use makes the surroundings clean and linked this to disease prevention.

*“Our surroundings used to be dirty with faeces from open defecation. Now most of us use latrines, the tree shades are clean and we use the place for community meetings”.* (IDI with 35-year-old male adopter).

Latrine use was also valued for creating a clean environment and being a means of preventing unpleasant smells. Some IDI and FGD participants stated that an unpleasant smell is perceived as being a cause of infectious diseases, with the strength of the smell reflecting the severity of the disease. For instance, participants stated that children’s faeces are less smelly, resulting in less severe diseases such as a common cold. Access to a latrine at night was thought of as an important benefit of owning a latrine, especially for children and sick or old people. On the other hand, some participants said that they still preferred open defecation to a latrine because they enjoy it more, though they did not practise it nowadays due to the lack of trees and bushes.

### Individual technology and contextual factors

As participants expressed during FGDs, people who had built a latrine in the past decided not to maintain or rebuild it because they had negative experiences, such as the collapse of their original latrine. Current latrine-owner households also expressed dissatisfaction with the existing pit latrines due to their poor strength and their inability to resist flood runoff and collapsing soil. People who had been replacing latrines were perceived as relatively better off economically. Participants identified durability, strength and depth as criteria for an excellent quality latrine. In one of the villages, taller latrines were preferred as more convenient for taller people. However, participants reported that quality latrines with good strength hardly existed in their community because construction materials were unaffordable, and almost all community members in Becho used unimproved pit latrines.

### Household-level factors

The sustainability of latrine ownership was influenced by the ability of households to construct a new latrine. Latrine-adopter households reported that most non-adopter households are from lower-income, older-age or female-headed households.

*"Can’t you see Mamo's mother? She does not own a latrine. She is poor and she doesn't have the capacity. Her elder son left her and now she lives alone. Do you think she can dig the soil by herself? Don’t you think that she should be assisted? Okay, for example you can also see Mimi's mother, or Mr. Abe Gofa. These people are poor. Do you think that they have the capacity? We force them to construct a latrine. Can they? These people need assistance"*.

(FGD with 42-year-old male adopter).

Similarly, latrine non-adopter households also perceived that they have less capacity compared with latrine-adopter households.

*“There is a difference between us and people who own a latrine. They own trees to construct a strong latrine, or they have a better income. But we use straw, grass or something like that to build latrines. Our latrine has been collapsing every year. Those who are better than us in terms of wealth did not face similar challenges like us”.*

(FGD with 32-year-old male non-adopter).

Limited availability of space in the compound after frequent replacement of collapsed latrines was identified as one of the barriers for sustainable latrine adoption.

*“All of us in this village are challenged. I have been building new latrines every year. Now I have finished the available space in my compound. This makes me feel very hopeless. I might stop building in the future”.*

(IDI with 35-year-old male adopter).

### Community-level factors

Lack of public latrines at the community level influenced people to practise open defecation while away from home. Women participants expressed that according to societal norms, women are expected to wash their hands after latrine use. There is a cultural belief that *injera* (local traditional food) baked by a woman who does not wash her hands after defecating can easily develop mould. Gender-related cultural norms strongly influence women to use latrines consistently because of the extreme shame involved if they are seen practising open defecation.

*"It is very challenging whenever we stay away from home. There is no public latrine anywhere. It is much more challenging for a woman. Sometimes I suffer from bladder pain as a result of holding my urine for a long time".*

(FGD with 17-year-old girl).

Theoretically, the government has designed post-triggering follow-ups to be carried out by people in one-to-five networks. However, neither the committee elected during CLTS nor the one-to-five networks took on the post-triggering follow-up role. As expressed during FGDs, the community did not set a collective norm against open defecation immediately after CLTSH-triggering, considering that they already had rules among one-to-five networks.

*"Rules that were set within one-to-five networks against OD in the village had never been implemented. Sanitation committee members that were elected during CLTSH triggering don’t have the power to implement those rules, since rules were set by one-to-five groups".*

(IDI …. Health extension worker).

Unaffordability and the economic conditions in the village were reported as major barriers for the adoption of improved latrines. It was inferred from the FGD participants that the average cost of low-quality traditional pit latrines is US $13 to $17, which is still perceived as too expensive by some poor community members. An improved latrine costs an estimated US $260 to $304.

### Societal-level factors

Climate and soil stability emerged as major challenges for sustained latrine adoption. As expressed by participants in IDIs and FGDs, the soil formation is spongy, holds too much water during the wet season and cracks during the dry season. Latrines constructed using local materials such as grass and straw failed to withstand soil and climatic conditions.

*"The soil in our surroundings is a dark black soil. It holds much water when it rains, and it cracks whenever it gets dry: that makes latrines collapse."*

(IDI with HH, 38-year-old male adopter).

However, the community has come up with some coping strategies to overcome latrine collapse, including building latrines under sturdy trees, digging a “V”-shaped pit, building latrines at the top of muddy ground, reusing water-filled latrines when they dry up, and casing the soil by inserting used tires. This last strategy, however, was perceived as unaffordable by most community members. Some participants mentioned adding salt to the water-filled latrines to dry them out more quickly.

Lack of local political commitment was demonstrated by inadequate budgeting for sanitation. Sanitation issues were left for the health sector to address. However, sanitation has received low priority within the health sector during programme planning, monitoring and reviews at the district, zone and regional levels. Within the health system, communication about the sanitation programme was very limited, being mostly only for reporting purposes.

*"Sanitation has been given the least priority in the health system. You can see the reporting system as an example; sanitation was not included in HMIS report. The issue of sanitation was raised at the end during the health programme planning at zone, maybe for five minutes, and it has never been properly addressed. Sanitation has never been raised during programme reviews. Less attention was given to sanitation both at the zonal and regional level compared with other programmes like MCH, HIV/AIDs/, Malaria, etc."*

(IDI, sanitation staff at the district level).

## Discussion

Most prior studies on sanitation focused on hardware and investigated individual- or household-level factors [10]. Based on the socio-ecological model and IBM-WASH framework analysis, the current qualitative study sought to explore the complex individual, social and environmental factors influencing the sustained adoption and consistent use of sanitation facilities in the rural Becho district of Ethiopia. Findings showed that several contextual, psychosocial and technology-related factors at multiple levels have influenced the sustainability of latrine adoption and consistent use of latrines.

The current findings showed that a high level of knowledge and preference for latrines was attained in the study area. A range of perceived health, social and economic benefits of latrine use was expressed by study participants. However, there was a perception of minimal health threat from children’s faeces, and limited or no hygienic child-faeces management. A study in India and one in Cambodia consistently reported similar findings in terms of people’s perception of child-faeces management [37, 38]. Even though open defecation practice has decreased remarkably over the past decade, the community did not move up the sanitation ladder, and there was very limited access to improved sanitation. Consistent with the current findings, other sources also reported low-level access to improved latrines. For example, the Ethiopian National Mini DHS in 2014 showed that only 8.0% of the population use improved latrines [39], while the JMP study reported a slight improvement to 28% in 2015 [40]. Despite these findings, sanitation programmes in Ethiopia still focused on expanding coverage, with less emphasis given to access to improved sanitation [15]. Better strategies are needed in addressing this issue.

At the societal level, the frequent collapse of latrines during rainy seasons due to poor latrine quality and soil conditions were identified as the key contextual and technology-related barriers to sustained adoption of any kind of latrine. While the use of locally available materials has been recommended for the sustainability of any adopted technology, the current study showed that latrines failed to be sustained because of the use of poor-quality local materials. Latrine reconstruction costs after collapse, the unavailability of space, the lack of a physically strong person in the family who has the capacity (labour) for digging the soil, and the inability to pay for labour costs all discouraged households from replacing their destroyed pit latrines and predisposed them to return to the practice of open defecation. Despite the national sanitation strategy, which specifies “no subsidies for household latrines” [16], findings in this study show that the poor were unable to replace latrines after frequent collapse, illustrating the issue of equity of access to improved latrines. This indicates that some flexibility may be needed with regard to latrine subsidy in Ethiopia. Evidence indicates that public subsidy has worked in other countries, although it failed whenever it did not take into consideration household preferences, addressing behaviour, or proper targeting [41]. Ethiopian national policy and sanitation strategy should consider the promotion of appropriate latrine technology based on contextual situations, including the environment.

Despite a high level of awareness and a positive attitude towards latrines, sustaining ODF status over time was a challenge linked to the poor implementation of CLTSH post-triggering follow-up and post-ODF activities. Despite the availability of a clear national strategy to scale up CLTSH all over rural Ethiopia and government commitments at the national level [16, 17], the lack of strong local leadership at the lower level negatively impacted its effectiveness, showing the need to strengthen local capacities. This suggests that large-scale CLTS scale-up should be planned cautiously, in line with the capacity and engagement of government structures at the lower level. Challenges related to post-triggering follow-up emerged due to the overlap of roles between the sanitation committee elected during CLTSH-triggering and the “Health Development Army (HAD)” or “one-to-five network”. Post-triggering follow-up needs to be integrated within these existing structures (HAD), with clearly defined roles. Capacity-building of these groups is also required in order to maintain the quality of CLTSH implementation.

Overall, the results of the study showed a lack of appropriate sanitation technology that can adapt to the environment, unhygienic child-faeces management and limited access to the poor as the main gaps that need immediate attention. Findings indicated that factors at different levels influenced each other. Sanitation policy formulation and implementation need to include demand creation for improved sanitation, financing options for the poor or sanitation-market development and engineering skill-building for latrine construction at the local level based on existing soil conditions. In Ethiopia, where the lack of access to improved sanitation accounts for more than 60% of the burden of disease in the country [7], the cost effectiveness of preventing diarrhoea and other sanitation-related diseases versus different investment modalities of providing access to improved sanitation is one of the priority research areas that we recommend for future studies.

Before generalizing our study’s findings, researchers must understand that the study was conducted among a very homogenous population with a similar ethnic and religious composition. The study’s findings can be generalized for a mostly rural Ethiopian context, except for factors related to soil and environmental conditions, which can apply to areas with a similar context.

### Strengths and limitations

The use of the SEM and IBM-WASH framework for exploring multi-level and multi-dimensional barriers is one of the strengths of the study. Stratifying latrine adopters and non-adopters enabled us to examine the problem from different angles and enhanced the credibility of our findings. The study team consisted of multidisciplinary experts, i.e., experts in environmental issues, public health and social sciences that enabled in-depth analysis of the research problem. However, subjectivity could not be completely ruled out due to the inherent limitation of the qualitative method. The findings may not be generalized to many settings, as the study was conducted only in a single rural *woreda*. Applying the IBM-WASH framework in the current study, we found several overlaps among the categories of psychosocial, technological and contextual factors. For instance, lack of appropriate latrine technology can be categorized under technology or under the contextual category. This was one of the challenges that we encountered during our study design and analysis. Although we were able to categorize individual-level factors into psychosocial, technology-related, and contextual dimensions, we were unable to follow this categorization for households to societal-level analysis. Although we conducted a total of eight FGDs, only one was conducted with each of the groups of adult women, adult men, female youths, and male youths, which limited us to conducting a subgroup analysis.

## Conclusion and recommendation

The use of the socio-ecological model and IBM-WASH framework helped to achieve a better understanding of multi-level and multi-dimensional barriers to sustained latrine adoption. The results indicate that there is a need to consider interventions that address multi-level factors concurrently.

## Abbreviations

CLTSH: Community-Led Total Sanitation and Hygiene
DHS: Demographics Health Survey
FGD: Focus Group Discussion
HEW: Health Extension Workers
IBM: Integrated Behavioural Model
IDI: In-depth Interview
NGO: Non-Governmental Organizations
OD: Open Defecation
ODF: Open-Defecation Free
SEM: Socio Ecological Model
TSNB: Theory of Social Norm Behaviour
WASH: Water, Sanitation and Hygiene
